# Coral reef aorta demonstrated by serial computed tomography: a case report

**DOI:** 10.1093/ehjcr/ytag732

**Published:** 2026-10-03

**Authors:** Tsuneaki Kawashima, Masaki Harada

**Affiliations:** Department of Cardiology, Tango Central Hospital, 158-1 Sugitani, Mineyama-cho, Kyotango, Kyoto 627-8555, Japan; Department of Cardiology, Tango Central Hospital, 158-1 Sugitani, Mineyama-cho, Kyotango, Kyoto 627-8555, Japan

**Keywords:** Coral reef aorta, Peripheral arterial disease, Computed tomography, Vascular calcification, Aortic stenosis, Case report

## Abstract

**Background:**

Coral reef aorta (CRA) is a rare vascular disorder characterized by dense, protruding calcified plaques within the abdominal aorta, often resulting in ischaemic symptoms. Because of its rarity and atypical presentation, CRA is frequently diagnosed at an advanced stage.

**Case summary:**

A 50-year-old male smoker with hypertension and hyperlipidaemia presented with progressive lower extremity symptoms. Four years before presentation, he developed bilateral lower limb numbness and was diagnosed with lumbar disc herniation, which likely masked the underlying vascular disease. One year before presentation, he developed intermittent claudication after ∼10 min of walking, followed by severe bilateral rest pain 1 month before presentation. The ankle–brachial index (ABI) was markedly reduced to 0.49 bilaterally. Computed tomography (CT) revealed extensive calcified plaques causing near occlusion of the infrarenal abdominal aorta. Serial CT over 6 years demonstrated progressive intraluminal calcification, facilitating diagnosis and treatment planning. Open aortic graft replacement with inferior mesenteric artery reconstruction was performed. Postoperatively, symptoms resolved completely, and the ABI improved to 1.13 (right) and 1.19 (left). At 1-year follow-up, the patient remained asymptomatic with the ABI of 1.07 bilaterally, and CT confirmed graft patency.

**Discussion:**

This case highlights the importance of considering CRA in patients with lower limb ischaemia, particularly when symptoms are initially attributed to non-vascular conditions. Serial CT is essential for detecting progression and enabling timely diagnosis. Open surgical intervention remains the most reliable treatment and can achieve excellent clinical outcomes in advanced disease.

Learning points

- Coral reef aorta may be misdiagnosed when coexisting conditions mask vascular pathology; serial CT imaging is essential for accurate diagnosis.
- Extensive circumferential calcification often precludes endovascular therapy, making open surgical reconstruction the preferred strategy in advanced disease.

## Introduction

Coral reef aorta (CRA) is a rare vascular disorder characterized by dense, coral-like calcified plaques protruding into the aortic lumen, most commonly affecting the juxtarenal or infrarenal abdominal aorta.^1^ Coral reef aorta has been recognized as a distinct clinical entity that can cause severe ischaemic symptoms, including intermittent claudication, rest pain, renovascular hypertension, and visceral ischaemia.^1,2^ Because CRA often presents in relatively younger patients and may mimic neurological or orthopaedic conditions, diagnosis is frequently delayed.^2,3^ Early recognition is crucial, as advanced disease may lead to life-threatening complications such as acute aortic occlusion.^2,3^

## Summary figure

**Figure:**
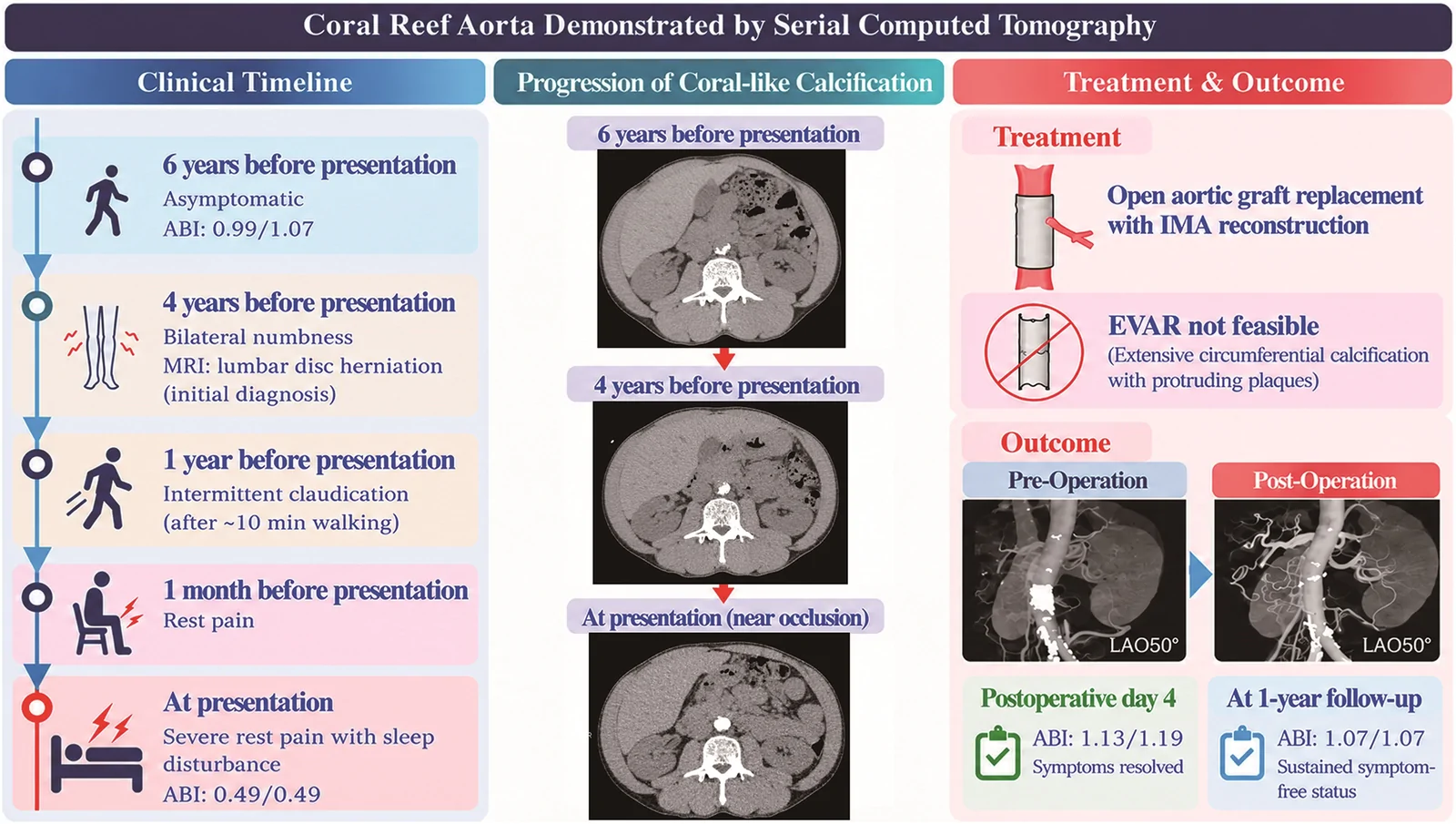


## Case presentation

A 50-year-old man with a history of smoking, hypertension, and hyperlipidaemia presented with progressively worsening lower extremity symptoms. Four years before presentation, he first noticed bilateral lower limb numbness and was diagnosed with lumbar disc herniation based on magnetic resonance imaging (MRI), for which conservative management was initiated. One year before presentation, he developed intermittent claudication after ∼10 min of walking. One month before presentation, severe bilateral rest pain developed, preventing sleep and prompting further medical evaluation.

On physical examination, his height was 166 cm and weight was 66.4 kg. Blood pressure was elevated at 173/115 mmHg. Femoral pulses were weak bilaterally, and distal pulses in the lower extremities were barely palpable. No skin ulceration or colour change was observed. Neurological examination revealed no focal deficits. The ankle–brachial index (ABI) was markedly reduced to 0.49 bilaterally, whereas previous measurements obtained 6 years earlier had been within the normal range (right 0.99 and left 1.07). Laboratory testing revealed severe hyperlipidaemia with a low-density lipoprotein cholesterol level of 225 mg/dL. Following the diagnosis, medical therapy was initiated for cardiovascular risk factor management, including hypertension and hyperlipidaemia.

Computed tomography (CT) of the abdomen demonstrated extensive, dense calcified plaques protruding into the lumen of the infrarenal abdominal aorta, resulting in near occlusion over a segment exceeding 4 cm in length (*Figure 1*). Non-contrast and contrast-enhanced images showed coral-like calcification predominantly arising from the posterior aortic wall. Review of prior imaging revealed progressive intraluminal calcification over a 6-year period, culminating in near-occlusion at presentation (*Figure 2*). These imaging findings were consistent with the characteristic features of coral reef aorta. Based on these findings, a diagnosis of CRA was established.

**Figure 1:**
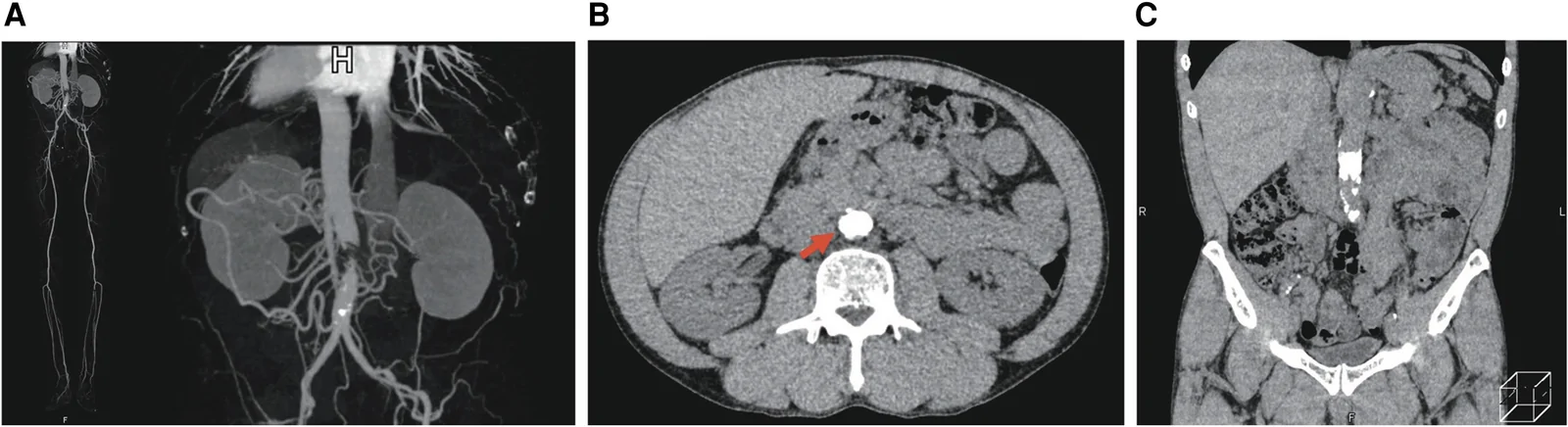
Abdominal CT at presentation. *(A)* Contrast-enhanced CT showing near occlusion of the infrarenal abdominal aorta. *(B)* Axial non-contrast CT demonstrating protruding calcified plaques within the aortic lumen. The arrow indicates a protruding intraluminal calcified plaque causing near occlusion. *(C)* Coronal non-contrast CT showing a segment of dense calcified plaque extending over more than 4 cm, resulting in near occlusion of the aortic lumen.

**Figure 2:**
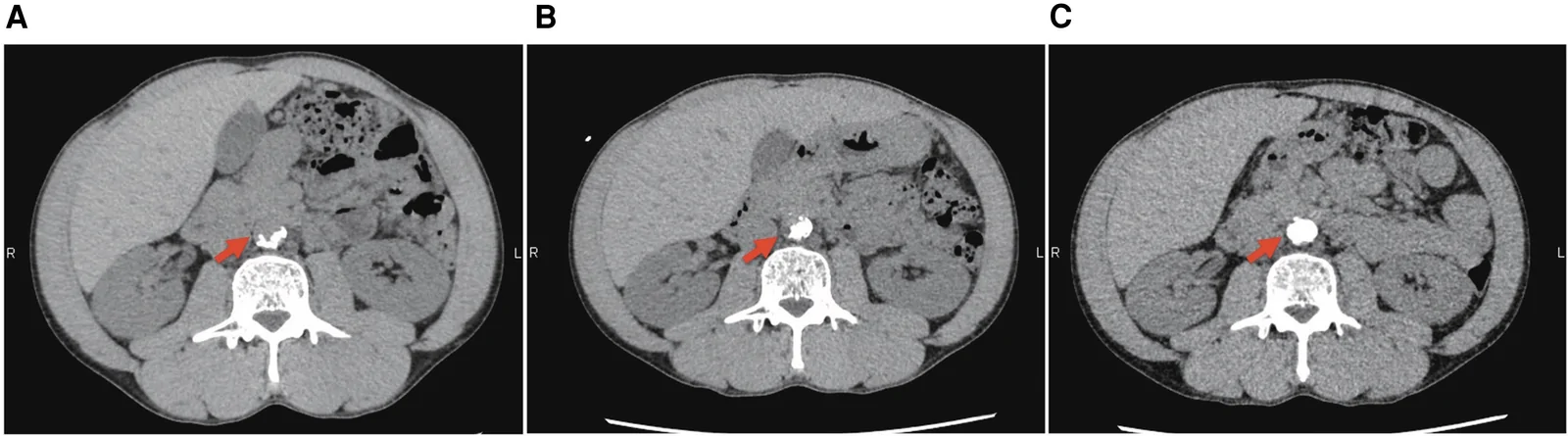
Serial abdominal CT images over a 6-year period. *(A)* Six years before presentation, calcification of the posterior aortic wall was observed. *(B)* Four years before presentation, the calcification had progressed and protruded into the lumen. *(C)* At presentation, extensive calcification over a long segment nearly occluded the aortic lumen. The arrows indicate intraluminal calcified plaques and progressive luminal narrowing over time.

Because of the extensive circumferential and protruding calcified plaques, the risks of distal embolization and inadequate stent expansion were considered high, making endovascular treatment unsuitable. In addition, the patient was deemed fit for open surgery, and therefore open aortic graft replacement was selected.

The patient underwent open aortic graft replacement from below the renal arteries to the proximal aortic bifurcation, with reconstruction of the inferior mesenteric artery (IMA) (*Figure 3*), 14 days after presentation. The postoperative course was uneventful. On postoperative day 4, the ABI improved from 0.49 bilaterally to 1.13 on the right and 1.19 on the left, with restoration of palpable femoral and dorsalis pedis pulses. Intermittent claudication and rest pain resolved completely after surgery, and the patient was discharged without complications. Postoperatively, medical therapy was optimized with pitavastatin (2 mg/day), ezetimibe (10 mg/day), sacubitril/valsartan (200 mg/day), and amlodipine (5 mg/day), achieving good control of blood pressure and lipid levels. Smoking cessation counselling was also provided as part of comprehensive cardiovascular risk factor modification. At 1-year follow-up, the ABI was 1.07 on both sides, and the patient remained symptom-free with sustained improvement in daily physical activity. Contrast-enhanced CT confirmed patency of the prosthetic graft and the reconstructed IMA.

**Figure 3:**
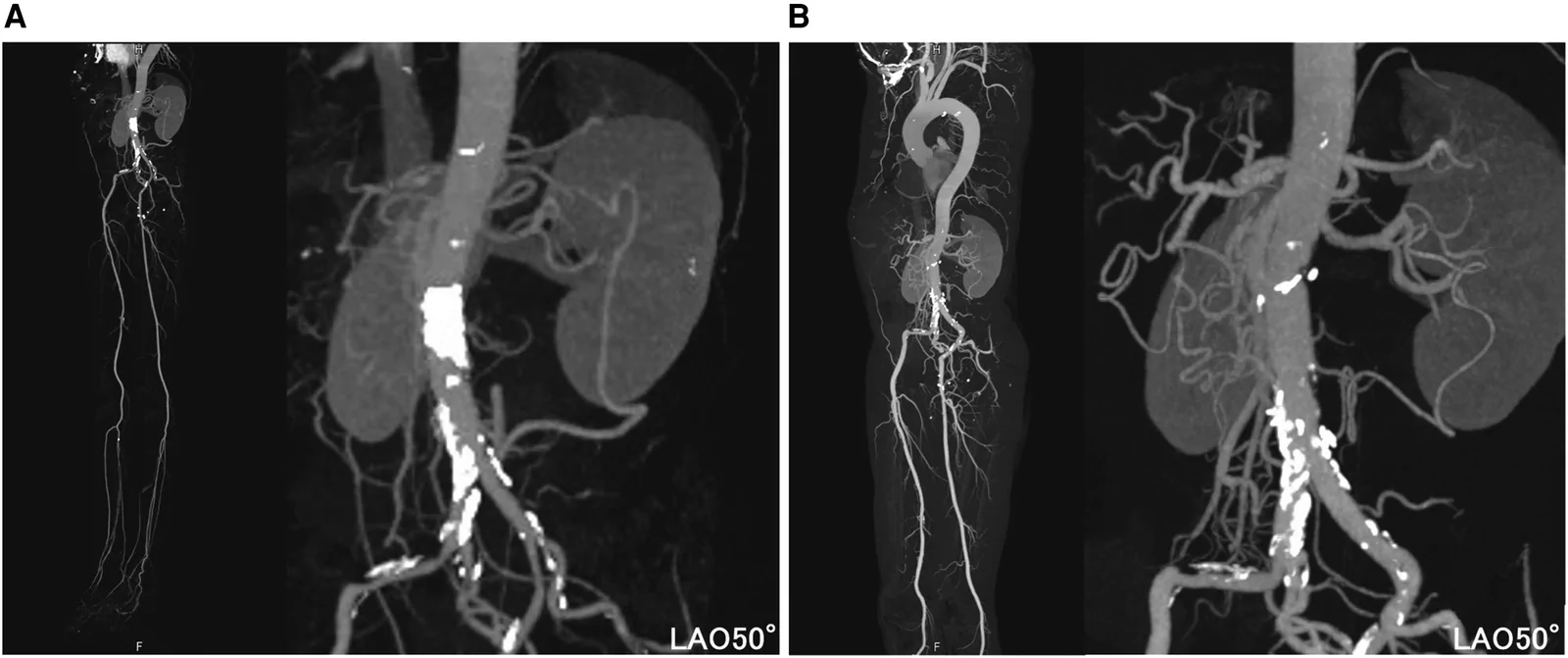
Preoperative and postoperative abdominal CT angiography (LAO 50° view). *(A)* Preoperative images showing near occlusion of the infrarenal abdominal aorta due to extensive calcified plaques. *(B)* Postoperative images demonstrating a patent aortic graft with restored blood flow.

## Discussion

This case highlights several important clinical aspects of CRA. First, CRA should be considered in the differential diagnosis of unexplained lower limb ischaemia, particularly when symptoms are bilateral, progressive, and disproportionate to peripheral arterial findings.^2,3^

In the present case, the presence of lumbar disc herniation on MRI findings (*Figure 4*) likely masked the underlying ischaemic symptoms, resulting in a delay in diagnosis, a pitfall that has been described in previous reports.^2,3^

**Figure 4:**
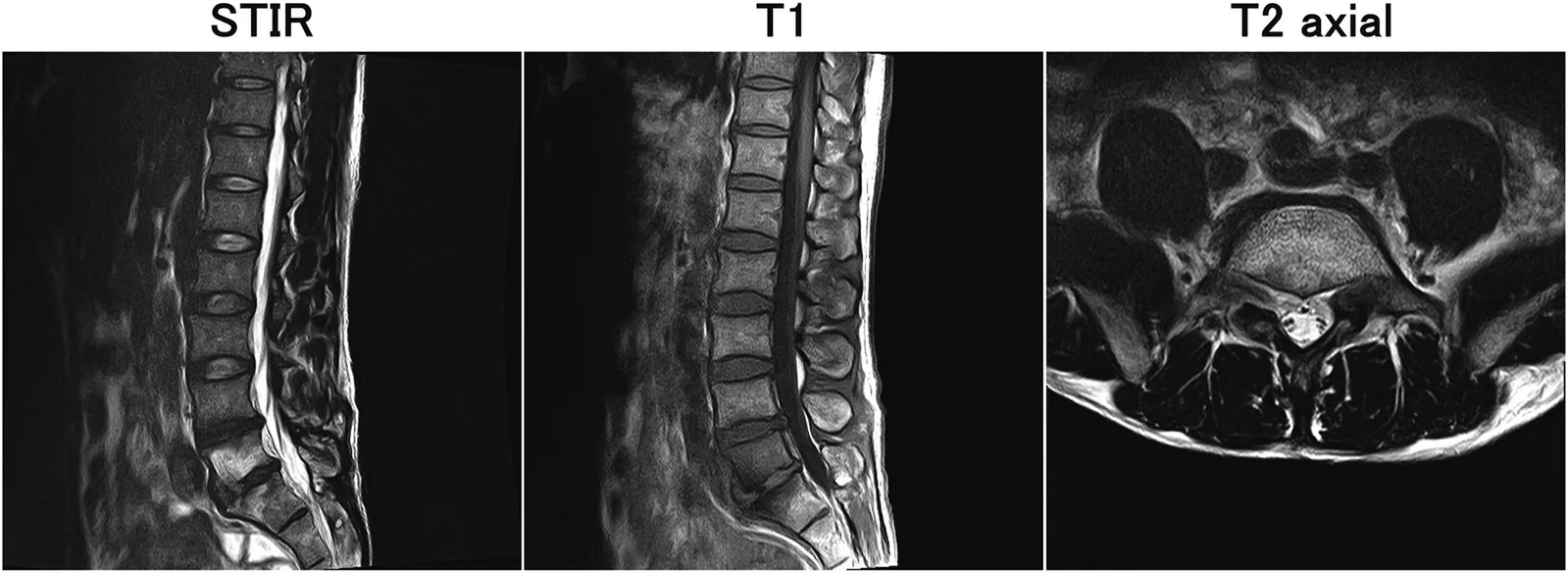
Lumbar spine MRI. Sagittal STIR and T1-weighted images, and an axial T2-weighted image, demonstrating disc degeneration and herniation at the L4/5 and L5/S1 levels.

Second, CT plays a pivotal role in establishing the diagnosis. It is particularly useful for delineating the characteristic intraluminal, coral-like calcification of CRA and accurately defining lesion extent.^4^ The availability of serial imaging over a 6-year period in this case provides valuable insight into the progressive nature of CRA.^2^ The presence of near occlusion over a long segment indicates advanced disease with significant haemodynamic impact, which has important implications for treatment selection.

According to the European Society of Cardiology (ESC) guidelines on peripheral arterial diseases, anatomical imaging is recommended when non-invasive testing indicates significant ischaemia or when symptoms are disproportionate to distal arterial findings.^5^ In patients with lifestyle-limiting claudication or chronic limb-threatening ischaemia, revascularization should be considered when symptoms persist despite optimal medical therapy.^5,6^

Regarding treatment strategy, open surgical reconstruction remains the most reliable approach for extensive CRA, as heavy calcification often precludes safe stent deployment and adequate luminal expansion.^2,3^ In previously reported surgical series, open repair has demonstrated favourable long-term outcomes with acceptable perioperative risk.^2,7^ In the present case, open aortic graft replacement resulted in complete symptom resolution and marked functional improvement, consistent with these findings. In contrast, endovascular therapy may be considered in selected patients with high surgical risk or limited disease extent; however, its feasibility is often restricted by severe calcification, which may impair adequate stent expansion and increase the risk of complications such as distal embolization or vessel injury.^8^ These anatomical features are well-recognized limitations of endovascular therapy in heavily calcified aortic lesions. Therefore, treatment selection should be individualized based on anatomical characteristics and patient risk profile.

Long-term postoperative surveillance after open repair should include clinical assessment for recurrent lower-limb symptoms, examination of peripheral pulses, and serial ABI measurements. Vascular ultrasonography or CT may be used as clinically indicated to assess graft patency, anastomotic complications, and progression of disease in untreated arterial segments. Continued cardiovascular risk-factor modification is also essential. In the present case, the patient remained symptom-free at 1-year follow-up, with normalization of the ABI and CT-confirmed patency of both the prosthetic graft and the reconstructed IMA.

The pathophysiology of CRA remains incompletely understood; however, disturbances in vascular calcification regulation have been implicated in disease progression.^9^ Given the potential for catastrophic complications, including acute aortic occlusion, early diagnosis and timely multidisciplinary decision-making are essential to prevent disease progression and serious complications, particularly in patients with rapidly progressive or bilateral symptoms.^2,5^ We believe this case provides clinically important insights into the diagnostic challenges and management of coral reef aorta.

## Lead author biography


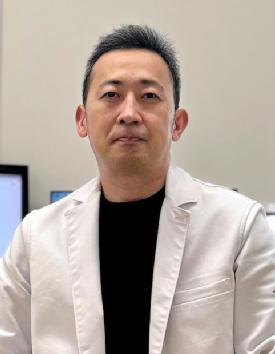


Tsuneaki Kawashima, MD, PhD, is the Head of the Department of Cardiology at Tango Central Hospital, Kyoto, Japan. His clinical interests focus on heart failure, cardiovascular imaging, and the diagnosis and management of cardiovascular diseases. His research background includes basic investigations into heart failure and myocardial metabolism at Kyoto University.

## Data Availability

The data that support the findings of this case report are available on reasonable request from the corresponding author.
